# Rocky milieu: Challenges of effective integration of clinical risk management into hospitals in Iran

**DOI:** 10.3402/qhw.v10.27040

**Published:** 2015-05-11

**Authors:** Jamileh Farokhzadian, Nahid Dehghan Nayeri, Fariba Borhani

**Affiliations:** 1 Department of Community Health Nursing, School of Nursing and Midwifery, Kerman University of Medical Sciences, Kerman, Iran; 2 Department of Nursing and Midwifery Care Research Center, School of Nursing & Midwifery, Tehran University of Medical Sciences, Tehran, Iran; 3 Department of Nursing Ethics, Medical Ethics and law Research Center, Shahid Beheshti University of Medical Sciences, Tehran, Iran

**Keywords:** Clinical risk, healthcare risk, clinical risk management, patient safety, quality improvement, clinical governance

## Abstract

Healthcare risks and clinical risks have been recognized as a major challenge in healthcare. Clinical risks can never be eliminated and can have serious adverse effects on patient safety. Thus, a clinical risk management (CRM) system has been introduced in the healthcare system to improve quality services. The aim of this study was to explore nurses’ experiences related to the challenges of implementing CRM in the organizational context. This qualitative study was based on the conventional content analysis of the Lundman and Graneheim approach, and it consisted of 22 interview sessions with 20 nurses. The purposive sampling method was used to choose the participants from three hospitals affiliated with the Kerman University of Medical Sciences. We used semi-structured interviews and review of relevant documents to collect data. The analysis of the data led to the emergence of “rocky milieu” as the main theme, and it consisted of three categories that, along with their subcategories, explain the challenges of implementing CRM. The three categories and their subcategories were (1) organizational culture and leadership challenges (decision and performance of leadership and cultural resistance to change), (2) limitation of resources (financial, human, and physical and equipment resources), and (3) variations and complexities in working conditions (the emotional, psychological, and social atmosphere and the heaviness of workload). Attempts have been made to establish CRM through clinical governance and accreditation, but organizational challenges have created a rocky milieu for implementing CRM. However, from an organizational context concerning the suitability of healthcare in Iran, there are obvious needs to move toward quality improvement and safe practices through the effective implementation of CRM.

In recent years, it has been recognized that healthcare involves some safety risks (Johnstone, Kanitsaki, Currie, Smith, & McGennisken, 2007). Healthcare risk or clinical risks have created concerns and challenges throughout the healthcare process (Verbano & Turra, 2010). In 2000, the Institute of Medicine (IOM) published a report entitled “To Err is Human: Building a Safer Health System” (Kohn, Corrigan, & Donaldson, 2000), and healthcare organizations began to focus on quality improvement using the healthcare risk management or clinical risk management (CRM) system (Franca, 2008). A CRM system is defined as administrative and clinical efforts to identify, reduce, and manage clinical risks (Adibi, Khalesi, Ravaghi, Jafari, & Jeddian, 2012). CRM can improve the quality of healthcare and help guarantee the safety of patients, visitors, and healthcare providers. It also can reduce the costs associated with healthcare systems and reduce the potential liabilities faced by employees (Verbano & Turra, 2010).

In other countries, such as the UK and the USA, progress has been made in CRM and in improving the safety of healthcare systems using approaches such as the clinical governance (CG) model (Specchia et al., 2010). These countries have started programs to implement CRM and to educate healthcare providers in hospitals (Leape et al., 2009). In 2010, the healthcare system in Iran began systemic efforts in this field, and the use of CRM was introduced as an essential principle of CG (Dehnavieh et al., 2013; Sheikhtaheri, Sadoughi, Ahmadi, & Moghaddasi, 2013).

A review of the literature showed that, despite the fact that 10 years have passed since the IOM's report, the rate of clinical risks is still high. Researchers in a study in the USA reported that the risk of harm to patients in hospitals was 10 times greater than had been estimated previously. In developed countries, approximately 10% of patients are harmed while receiving care (Groves, Meisenbach, & Scott-Cawiezell, 2011; Vincent, Moorthy, Sarker, Chang, & Darzi, 2004), and 5–13% of them die as a result of clinical incidents (Adibi et al., 2012). The potential risk of harm to patients in developing countries, such as Iran, is much greater than the risks in the developed countries (WHO, 2014). Research in an Iranian hospital reported that at least 3.6% of patients had experienced clinical incidents and that 5.3% of all patients’ deaths had been due to clinical incidents (Adibi et al., 2012). These risks imposed billions of dollars of costs to the healthcare system (Vaismoradi, Salsali, Turunen, & Bondas, 2013).

Because clinical risks can never be totally eliminated (Briner, Kessler, Pfeiffer, Wehner, & Manser, 2010), and because of their complexity and seriousness, implementing CRM is considered to be one of the most important missions of healthcare organizations (De Vries, Ramrattan, Smorenburg, Gouma, & Boermeester, 2008). Despite international concerns and efforts, healthcare is not moving quickly enough toward the implementation of effective CRM, and nurses face many challenges in their efforts to implement CRM. In a study in Canada, researchers reported that additional research is needed to understand the factors leading to unsafe practices, to define the challenges associated with eliminating or reducing these factors, to acquire additional assessments of social and cultural factors, and to assess their impacts on the healthcare delivery system (Espin, Lingard, Baker, & Regehr, 2006).

In Iran, some studies have been conducted concerning the various aspects of a CRM system, such as the safety culture, nursing errors, and the prevalence of clinical incidents. These studies indicated that the status of CRM is not appropriate and that Iran's healthcare system still needs significant improvements to achieve international standards for patient safety (Adibi et al., 2012; Baghaee, Nourani, & Khalkhali, 2012; Sheikhtaheri et al., 2013). Ravaghi et al. reported that important factors in implementing CG and CRM are (1) understanding challenges from the viewpoints of healthcare providers and (2) resolving them so that different quality programs can be implemented in healthcare (Ravaghi, Heidarpour, Mohseni, & Rafiei, 2013).

According to the experience of the first researcher who was responsible for the implementation of CG, there are weaknesses in implementing CRM. In addition, there are ambiguous and unknown challenges in the implementation of CRM that resulted in the patients’ negative attitudes, complaints, and dissatisfaction concerning physicians’ and nurses’ performance. Two studies conducted at hospitals in Iran indicated that the extent of dissatisfaction and the number of complaints are increasing. Many of the complaints about nurses and physicians resulted from their perceptions of the clinical risks (Hedaiati, Nejadnik, & Setareh, 2012; Jabbari, Khorasani, Jazi, Mofid, & Mardani, 2014).

Obviously, despite the great efforts that have been made by hospitals to implement quality improvement approaches and establish the principles of CG, particularly CRM, there are some obstacles and gaps in the healthcare system. Exploring and identifying organizational and human challenges using qualitative studies help hospitals to be aware of their deficits in CRM so that the problems can be removed and standards of patient safety can be implemented and promoted. We found no comprehensive, qualitative study exploring the challenges of implementing CRM from the nurses’ perspective in Iran. It is worth noting that implementation of the CRM program is one of the most important responsibilities of nurses. Therefore, it is essential to identify and understand the challenges in the implementation and integration of CRM in the organizational process in hospitals from the nurses’ perspectives. In addition, the implementation of CRM has been characterized as a complex challenge by healthcare professionals in hospitals, and qualitative studies are required to explore the anticipated complexities (Groves, Finfgeld-Connett, & Wakefield, 2012). This qualitative study was conducted to explore nurses’ experiences with implementing CRM in teaching hospitals in Kerman, Iran, and the organizational challenges they faced. Kerman, with a population of more than 722,000, is the largest city in southeast Iran, and its hospitals are actively implementing CRM system.

## Materials and methods

### Research design and setting

This study was part of a larger study conducted to explore the CRM process in nursing. In this part of the study, a conventional qualitative content analysis method was used to explore and identify participants’ experiences with the organizational challenges of implementing CRM. In this study, nurses were interviewed at the three teaching, referral, and physical-care hospitals associated with Kerman University of Medical Sciences.

### Ethical considerations

The study was conducted in accordance with the Declaration of Helsinki. First, approval for the study was obtained from the ethics committee affiliated with Kerman University of Medical Sciences (Medical Ethic No: 93/145). Then official permission for collecting the data was obtained through communication between the director of Razi Nursing and Midwifery school and the hospitals’ nursing directors. The objectives of the study were explained to the participants, and they were assured that they could withdraw from the study at any time if they wanted to do so. Each participant signed an informed consent form. In addition, the confidentiality of personal information and of the interviews was assured. They were informed that the interviews would be recorded for ease of transcription and that the audio files, with no names associated with them, would be kept in a safe place.

### Participants and sampling method

The purposive sampling method was used to select participants in this study. The inclusion criteria included: (1) willingness to participate in the study, (2) being in a good physical and emotional status, (3) experience of more than 1 year in the CRM field, and (4) having at least a BS degree and some practical experience about integrating CRM into workplace. The first researcher went to different wards and, after mentioning the objective of the study, she invited the participants and scheduled an interview date. The sampling was based on the principles of maximum variation to capture a vast range of perspectives and experiences. Therefore, 20 nurses with diverse backgrounds in sex, age, years of work experience, type of employment, type of ward, and position (including matron, risk manager, clinical supervisor, educational supervisor, head nurse, officer of quality improvement, and clinical nurse) were enrolled. The participants were continuously enrolled until data saturation was obtained.

### Data collection

In this study, methods of data collection were face-to-face, semi-structured interviews, field notes, and reviewing and reading of relevant documents. The first researcher conducted all the interviews in Persian, which were then translated into English. Each interview began with topic guide questions; for example:

“Would you please describe the clinical risks you have been faced with while caring for patients?”, “Would you please describe your experience of clinical risk management?”, “Would you please describe the conditions or experiences in caring for patients at risk?”, “How did you manage the situation?”, “How was the risk created?”, and “What barriers existed to managing these risks?” Then, for deepening the interviews, clarifying the participants’ responses, and covering the research objectives, directive and exploratory questions were asked: “Would you explain the situation clearly?” Interviews were performed at a predetermined appointment and in a relaxed and appropriate atmosphere in a hospital setting. On average, each interview lasted from 35 to 65 min. Information was saturated after 20 interviews and two additional interviews were conducted to ensure data saturation. To supplement and clarify the information in the first interview, two participants were interviewed twice.

In the hospitals, there were numerous documents about CRM such as forms of incident reports and root cause analysis, along with protocols and guidelines, etc. The documents were used to complete the data from the interviews and field. Therefore, the researcher asked some questions about the role and application of documents for managing clinical risk: “Would you share with me your experiences about using documents such as high-risk drug injection protocol for managing clinical risk?”, “How do you encounter barriers for applying clinical guidelines such as safety of blood transfusions?”, and “How can these documents facilitate clinical risk management?”

### Data analysis

In line with the purpose of the study, the data were collected and analyzed simultaneously by the conventional qualitative content analysis method by Lundman and Graneheim (Elo & Kyngäs, 2008; Graneheim & Lundman, 2004). Thus, based on coordination and allowance of the participants, the whole interviews were recorded by a digital audio recorder and then converted into audio files on computers and transcribed word by word. All the transcribed texts from the interviews and the texts from the field notes were regarded as the unit of analysis. For immersion in the data and gaining insight into and understanding about the entire process that took place, each text was read and reviewed several times. Then, the meaning units were named in terms of their latent and manifest contents using open codes. These codes were based on the similarities, and their differences were categorized under more abstracted labels. The data reduction process helped to better describe and understand the phenomena. The abstracting process was continued until all of the themes were extracted. MAXQDA software (version 10) was used to facilitate the classification process and continue data comparison.

#### Trustworthiness

Strategies for enhancing the trustworthiness of the findings were based on Lincoln and Guba’ framework. Therefore, credibility, transferability, dependability, and confirmability were considered to ensure the trustworthiness of this research (Graneheim & Lundman, 2004; Long & Johnson, 2000). To enhance their credibility, the researcher established a friendly relationship with the participants and had prolonged engagements with the research settings (in the 9-month period from January to September 2014). To increase the breadth and depth of information, data collection was conducted using different sources (interviews, review of documents, and field notes). Summaries of the interviews and results were returned to the participants to confirm that the current findings reflected their experiences and perceptions (member checks). In addition, the codes and methods of categorization were continuously reviewed by the research associates (peer check). The encoding process and access to categories were approved by some of the faculty members specializing in qualitative research also (external check).

To achieve conformability of the data, the research processes and the decisions made during these processes were recorded accurately and reported in case others might wish to follow and audit the research results. In order to increase the transferability of the present findings to other locations or groups, sampling was designed based on maximum variation in terms of sex, age, years of work experience, type of employment, type of wards, and position of nursing. In addition, to describe the context of the study, information about the participants was provided. Direct quotations were used to support themes, so readers can judge whether the findings reflect the participants’ perspectives or not.

## Results

There were 20 participants in the study, which included 14 women and 6 men, aged 32 to 46 years old, with the work experience ranging from 2 to 25 years. Five participants had master's degrees and the 15 others had bachelor's degrees in nursing. The nurses who participated included a matron, a risk manager, two clinical supervisors, an educational supervisor, two head nurses, two quality improvement officers, and 11 clinical nurses. Two of the participants were committed employees,^1^ two others were contract employees,^2^ and the others were all formal employees.^3^ The majority of the participants had worked in several wards.

## Rocky milieu

From the analysis, the main themes of “rocky milieu” emerged to describe the challenges of implementing and integrating CRM in healthcare. The main concern of nurses consisted of three categories (Table I) and the description of the categories illustrated the health system challenges in Iran for quality and safe care using CRM. Organizational milieu was generally not suitable, because factors such as organizational culture and leadership challenges, limited resources (financial, human, and physical and equipment resources), and varied and complex working conditions had created negative perceptions about the organizational context.

**Table I:** Categories and subcategories conceptualizing rocky milieu: challenges to implement of CRM

Main theme	Category	Subcategory
Rocky milieu	Organizational culture and leadership challenges	• Decision and performance of leadership • Cultural resistance to change
	Limitation of Resources	• Financial resources • Human resources: - Inadequate productivity - Recession of empowerment - Inappropriate evaluation of performance - Lack of motivation - Personal characteristics and professional competencies - Ethical and professional commitments • Physical and equipment resources: - Medical equipment, medical facility, and mechanical supplies - Technology - Physical and sanitary environment
	variations and complexities in working conditions	• The emotional, psychological, and social atmosphere • Heaviness of workload

## Organizational culture and leadership challenges

This category consisted of two subcategories of “decisions and performance of leadership” and “cultural resistance to change.”

### Decision and performance of leadership

According to the experiences of the participants, leaders and the governance team had to move all the resources and culture of the organization for successful implementation of CRM. Their actions and provision of a strategic policy would serve as a roadmap to ensure the successful implementation of CRM. But, the leadership behaviors of managers, such as poor quality of supervising and controlling, poor management of resources, inconsistency of management, lack of proper management style, and ineligible leaders, were among the CRM implementation challenges that were reported based on the experience of the participants. One of the clinical supervisors stated the following point concerning the failure of the supervisory staff:

One manager only puts a tick mark in the checklist of round management. However, s/he does not have any risk management perspective. Another manager has such a perspective, but s/he does not precisely put a tick mark in the check list; there is no coordinated protocol for supervision.

A clinical nurse stated: “Some managers look like police and are always looking for non-compliance, instead of compliance.”

In addition, inadequate attention of different levels of managers to improvement plans, excessive attention to formal and futile documentation, and lack of support from staff were mentioned as other challenges in CRM programs. One clinical nurse said:

Managers only ask us about documents. Mental involvement with documents has made us neglect patients; it is true that documentation is important, but it is more like futile and false paperwork.

The data showed that most of the decisions and performance of different levels of leadership suggest that they do not have a clear and explicit understanding about basic principles, administrative aspects, and CRM requirements. A matron stated:

There is no support where there should be. We intend to institutionalize CRM systems in hospitals, but we notice that our high-ranking managers are not familiar with the system at all, do not move in line with us, and do not know the system's requirements.

### Cultural resistance to change

According to the participants’ experiences, cultural change was necessary for the implementation of CRM. But, there was resistance to this change. A risk manager said:

Death of a system happens when it is satisfied with itself and believes that it is fine and does not need any changes.

Concerning resistance to change, one of the clinical supervisors said:

There is always resistance to preparing a clinical guideline such as manner of suction, which is prepared for the ease of nurses. They have excuses like they do not have enough time and there are too many patients, etc.

Participants considered the safety culture as the most important cultural change. They thought the slow movement to the safety culture was the biggest cultural challenge that had adversely affected the optimized role of nurses. A head nurse said:

In our meetings, managers ask us to brainstorm about the errors and patient safety incidents; but, they will resist any ideas which are in contrast to the ideas of some people. Safety culture has not been adopted; they still have traditional, rather than systemic, approaches to problems.

Another challenge of nurses concerning the safety culture was fear of blame and punishment. One of the clinical nurses said:

When an error happens, I do not report it, because I do not want to lose per-case and have reduced evaluation score. Due to lack of culture-fostering, when talking about errors, those issues become valuable.

## Limitation of resources

Implementation of CRM involves management of “financial,” “human,” “physical,” and “equipment” resources, but limitations and shortcomings exist in these resources.

### Financial resources

The participants, especially directors, considered financial resources as an important prerequisite for the implementation of CRM. But, lack of financial resources has led to the ineffectiveness of CRM programs. A nurse responsible for the quality improvement office said:

For several years we have written action plans for improving manpower, equipment, physical space, etc. But, each year is the same as the last one; it is left incomplete due to lack of budget. When there is no budget to fix the structure, it is obvious that no improvement will occur in caring.

### Human resources

According to the participants’ experiences, efficient and competent human resources are effective in achieving the objectives of a CRM system. But, human resource management is also accompanied by many challenges. The challenges were described in the following six dimensions:

#### Inadequate productivity

Experiences of the participants indicated that various factors, such as lack of staff, especially hired and competent ones; use of inexperienced, contractual, and committed (temporarily employed) staff; inappropriate staffing; and aggregation of experienced staff on morning shifts have caused exhaustion and inadequate productivity of staff and thus influenced the implementation of CRM activities. A clinical supervisor stated:

When managers do not have the power to provide manpower, how could they implement CRM? Two nurses are responsible for 25 infected, elderly patients on a shift; each of these patients needs special care. How should we expect these personnel to implement risk management? About 70–80% of errors are caused by fatigue and lack of personnel.

#### Recession of empowerment

Review of the documentation indicated that educational programs were held on different aspects of the CRM system and safety in order to empower all the staff and foster cultures for integrating CRM in practice. Moreover, supervisory and clinical auditing as well as dialogue among participants with the aim of learning from errors and conducting its analyses was used as proactive education. In spite of these measures, nurses’ experiences suggested that managers acted very weakly in the empowerment of human resources. Educational programs did not sufficiently empower employees to participate in the implementation of CRM and some of the challenges undermined the effectiveness of teaching. Concerning taking classes, work conditions, and inappropriate educational evaluations, a nurse stated:

Most of the educational classes are held in the morning. Because we only have one month off, we have to attend these classes during our working hours; sometimes we cannot because of having crowded wards. Empowerment classes and tests are only formalities; we only want to fill out a test sheet and get a score and degree.

About lack of resources, educational supervisors said:

We know that we should use other methods for teaching in most places and these traditional methods are not appropriate; however we really do not have the resources and experienced people.

#### Inappropriate evaluation of performance

Although nurses expressed that the performance evaluation was one of the important techniques that can be used to empower employees in terms of achieving the CRM objectives, several factors could cause dissatisfaction and apathy among the staff in terms of implementing CRM activities. These factors included lack of an appropriate system for rewarding and punishing employees and unfair evaluations. A head nurse stated:

Our approach in the personnel evaluation is not based on selecting the competent and empowerment. Another nurse said: Evaluation is all prejudice; the personnel who work well, show interest in patients, and report errors are not different from the rest.

#### Lack of motivation

According to the data, lack of motivation caused indifference and dissatisfaction among the employees, whereas the implementation of CRM requires motivated and energetic employees. Spiritual and material motivations and other encouragements can enhance nurses’ satisfaction and participation in CRM activities. In this case, a quality improvement officer said:

There are no financial and spiritual incentives; wage and overtime payment of the staff are not sometimes paid on time. Nurses are unmotivated and consider risk management activities as additional work and without pay which is beyond their duty.

#### Personal characteristics and professional competencies

The physical, psychological, and personality characteristics of the staff had a tremendous effect on the CRM implementation. A risk manager said:

Some personnel have special personality types which do not let supervisors warn them about their errors: they are bad-tempered, disturb the shift work and concentration of other personnel; these issues increase the clinical risks.

In addition, the participants’ experiences revealed that managers must ensure having skillful, competent, experienced, and knowledgeable personnel in order to succeed in the implementation of CRM. However, in this case, nurses may suffer from many faults and shortcomings. A nurse with 19 years of experience expressed:

Both nurses of the ward were novice and unskilled; they injected the wrong dose of magnesium sulfate to the patient and the patient died.

#### Ethical and professional commitment

Hospitals need good-tempered and committed staff. Sometimes, lack of commitment and accountability cause negligence, injury to patients, and failure of CRM programs. One of the supervisors stated:

The personnel responsible for the patient does not pay attention to the patient's conditions which are not stable and transfers the patient to ward 2. There, the personnel still do not pay attention that the patient is not in good conditions, do not quickly accept him, and neglect the patient. After 10 min, they notice that the patient has died. It is lack of accountability!

## Physical and equipment resources

Implementation of CRM requires physical assets and extensive equipment. However, from the participants’ perspective, limitations in terms of resources have made the implementation of CRM very difficult for nurses. This challenge could be divided into three important dimensions:

### 

#### Medical equipment, medical facility, and mechanical supplies

Regarding unsafe, old, and non-standard equipment; lack of equipment, medical, and facility supplies; increased workload; risks; and low quality of services; an emergency nurse stated:

The main things that are more harmful for patients are lack of beds, equipment, manpower, and space. A patient with heart problem refers to the hospital, but all beds, even the extra, are full. We have to hospitalize the patient in the cold hallway without a monitor on the stretcher; this situation is really dangerous for the patient. They need facility and equipment more than anything else.

#### Technology

The participants stated that many technologies, such as IT technology, could empower staff in clinical care and decision making. However, many of the new technologies, along with the required hardware, were not available in hospitals. Many staff members did not have the knowledge and skills to use such technologies. One of the head nurses said:

We do not have access to comprehensive and reliable information. We do not have an organized electronic system for managing safety information of patients, nor decision-making support systems.

#### Physical and sanity environment

The participants believed that other factors led to the burnout of physical and mental capabilities, reduction in the quality of care, and increased clinical risks. These other factors included an unhealthy environment, lack of physical space, non-standard architecture, number of patients which exceeds the capacity of the ward, high levels of noise, poor lighting, and unsafe work environment. A clinical nurse stated:

Screen building in emergency department is very old and inappropriate; if a patient arrests on bed number 6, we cannot take the equipment to the patient and resuscitate him/her from both sides. This building has a very improper and small space, which increases our workload.

Review of documentation indicated: “Status of the separation, collection, and disposal of hospital waste was inappropriate. This condition was accompanied by poor hand hygiene, which threatened the safety of patients and staff.”

## Variations and complexities inworking conditions

### Emotional, psychological, and social atmosphere

Participants’ experiences suggested that a suitable and efficient atmosphere can affect the professional growth, empowerment, and productivity of employees and increase the quality and safety of their work. One of the clinical supervisors stated:

Today, we need a supportive and delegated environment; growth occurs in such a proper environment.

However, factors such as unprofessional interactions with clients and colleagues and nurse–physician inappropriate relationships create an unfavorable emotional, psychological, and social atmosphere.

A clinical nurse said:

Some doctors are too impatient. One day we had a very bad hemothorax in the hospital and we had to call the doctor to the hospital in clinic hours. He insulted us in front of the patient and his family; how can that patient's family trust us?

In addition, various factors have led to a poor working environment, such as anxious and angry companions and families of patients, stress, tension, and risk-prone environment. For example, an emergency nurse said:

Companions of the patient who had died began to offend us and break down the equipment; they wanted to beat the personnel. Well, nurses have to work in this kind of environment which is full of stress and anxiety and their lives are in danger.

### Heaviness of workload

Experiences of the participants and field notes of the researcher indicated that various factors increased the workload of nurses, including complex and high-risk situations; lack of time; time-consuming nature of error reporting; shortage of nurses, especially on night shifts; high number of patients; and increased volume of documentation and paperwork, which could result in decreased communication with patients and inadequate care for their needs and make the work conditions even more inappropriate. An emergency nurse stated:

Workload is high; some of the patients are not noticed in bustle. Sometimes, a very unwell patient comes, but we are so busy with others that neither a doctor nor a nurse can see the patient. Suddenly, we understand by the noise of a companion that the patient has arrested.

Sometimes exhaustion and heavy workload lead to actions that are not morally acceptable: low-quality work, using sick leave, or absence from work. These practices can establish a vicious cycle and put additional pressure on other colleagues, which could even deteriorate the shortage of manpower. (field notes)

## Discussion

In this study, some insights were gained into the organizational challenges of CRM system in Iran by exploring nurses’ experiences. The main theme derived from the analysis of the data was the “rocky milieu,” which consisted of three categories: (1) organizational culture and leadership challenges, (2) limitation of resources, and (3) variations and complexities in working conditions. These results suggested that nurses are expected to provide safe and high-quality care through the implementation and integration of CRM in healthcare. But, an appropriate organizational milieu is not provided in hospitals to achieve safe care using this system. The mentioned organizational challenges have created a rocky milieu for the implementation of CRM, showing that hospitals are far from achieving optimum, or even safe, conditions. Given that, the ultimate aim of healthcare providers is safe passage of clients from the care and treatment process. However, this organizational milieu is unable to protect the health and well-being of patients in their travel through the healthcare system.

In this work, the categories of culture and leadership were among the major challenges in the implementation of CRM. Other studies have also reported different levels of dissatisfaction with the organizational culture and challenges for the implementation of quality improvement programs, such as CG, in Iran and other countries. Although organizational culture has many dimensions, unwillingness to change and weaknesses in safety culture have been reported as the important challenges in developing this program (Chiozza & Plebani, 2006; Dehnavieh et al., 2013). Dehnavieh, et al. reported that inefficient leadership, lack of commitment of leadership, and cultural development had adverse effects on communication and cooperation among employees. Therefore, these factors could result in significant challenges for quality improvement programs in many cases (Dehnavieh et al., 2013). In another study, the factors related to the organization, such as weakness of a safety culture, existence of a culture of blame, lack of supporting staff, disproportionate reactions and performance of managers, and absence of a mood of teamwork were considered as the barriers to reporting errors (Hashemi, Nikbakht, & Asghari, 2011b). Despite the complexity of organizational factors, such as culture, structures, and processes, change in the organizational context is possible, but it will not be easy. Thus, it is essential to identify potential strategies for change (Krein et al., 2010).

One of the other main categories in the present study was the limitation of human, financial, and physical resources. In parallel with this study, several studies have reported various aspects of human resources as challenges to the implementation of quality improvement systems and safety programs, which include lack of competent personnel and lack of motivation (Dehnavieh et al., 2013; Nicklin & McVeety, 2002; Ravaghi et al., 2013; Vaismoradi et al., 2013), disproportionate number of personnel with the required skills and experience (Nassiri, Haghshenas Kashani, & Rabiee, 2010), lack of knowledge and skill, and low accountability and commitment (Hashemi et al., 2011; Ravaghi et al., 2013), and defects and shortcomings in educational and empowerment programs (Briner, Manser, & Kessler, 2013; Sujan, 2012). In a qualitative study in the UK, the role of human resources in the implementation of processes to change the culture of a workplace was considered to the crucially important. But, limited financial resources for human resource management could cause problems with the CRM implementation (Som, 2007).

These results indicated that many managers are still not prepared to take risks in order to inject funds to provide the infrastructure of human resource management (Gangi Zehi, 2011). Therefore, in hospitals, planning for empowerment and improvement of human resources should be placed in a strategic program. Educational programs should be correct, principled, based on the needs of nurses, in line with the purposes of CRM, and continued constantly. Employees’ performance evaluations should be based on the principles of CG. To improve performance, feedback should be given to employees, all of their evaluation documentation should be made available to them, and the documentation should be used in the employees’ empowerment process, for example, for designing appropriate educational programs (Hooshmand, Tourani, Ravaghi, & Ebrahimipour, 2014).

In terms of financial and physical resources, such as medical technology and equipment, the results of this study were similar to those of the previous studies. The researchers in those studies have also reported that lack of resources, especially human, financial, and time, and inappropriate design of information technology are the main obstacles to implementing quality improvement programs, patient safety, and incidence reporting (Krein et al., 2010; Ravaghi et al., 2013; Vaismoradi, Bondas, Salsali, Jasper, & Turunen, 2014). Development of physical and facility resource infrastructures as a strategy is significant for improving the safety and effectiveness of the CRM system.

Another important concern or challenge in the category of physical resources and equipment was the physical environment. In some studies, physical environment is not as clearly separated as it was in our study, but in general, equipment, technology, and resources have been mentioned as the main environmental factors (Aiken, Clarke, Sloane, & Sochalski, 2001; Squires & Juárez, 2012; Vincent et al., 2004). This difference is probably because in the hospitals in Iran, the problems that threaten patient safety in the physical environment are very evident and clear and people tend to judge the quality of services based on the appearance of the physical environment. This issue is in parallel with the results of the literature review that indicated that the status of hand hygiene (Najafi Ghezeljeh, Abbas Nejhad, & Rafii, 2013) and the status of separation, collection, and disposal of hospital waste are not appropriate in hospitals and there are various problems that require more attention than this issue, including use of new methods for the safe disposal of hospital wastes (Bazrafshan & Kord-Mostafapoor, 2010).

Another major category was the variations and complexities of the working conditions. In this regard, two studies have reported inappropriate factors of process characteristics, duties, working conditions, workload, and fatigue in environment as negative factors in the implementation of CRM and patient safety programs (Brady, Malone, & Fleming, 2009; Tang, Sheu, Yu, Wei, & Chen, 2007).

The organizational milieu in which nurses provide care affects working relationships, quality of care, patients’ safety outcomes, job satisfaction, stress, and desire to change. For the development and implementation of CRM, a suitable context must be provided for nurses by their leaders. When the organizational context is improved, employees will be more satisfied and have a greater tendency to implement and integrate CRM in practice. Favorable working conditions, safety, and physical and mental health will be improved for patients and staff. Leaders should understand nurses’ concerns, such as heaviness of workload and limitation of resources, in order to implement CRM. By creating a positive and proper atmosphere and providing support and encouragement, leaders can improve the possibility of significant positive changes, including implementation of CRM. Nurses also should try to create a safe emotional, mental, and social environment and promote and develop their personal and professional growth in this environment.

### Limitations

This qualitative study explored the challenges of implementing CRM in only three teaching hospitals in one area of Iran. Therefore, the transferability of findings from qualitative work should be considered with caution. A more comprehensive profile and insight into CRM requires the inclusion of different hospitals in terms of specialty, ownership, geographical status, and cultural and environmental conditions. Such a survey can lead to national strategies for improving CRM and identifying challenges that may not have been captured by the limited scope of this study.

## Conclusions

In this study, we explored the challenges of organizational CRM from the perspectives of nurses who have experienced it. The findings indicated that, despite emphasis on the establishment of CRM in CG and accreditation approaches, hospitals in Iran are not moving sufficiently toward high-quality, safe practice. The organizational context was not prepared suitably, and organizational challenges have created a rocky milieu for effective integration of CRM in healthcare. Studying organizational challenges based on in-depth, comprehensive information is an important step in hospitals to develop CRM. We suggest additional quantitative and qualitative research to assess the contributing factors of CRM, to identify the facilitators and barriers, and to develop interventions for improving CRM.

## Footnotes

1 “Committed employees” work temporarily and for a specific duration.

## Footnotes

2 “Contract employees” generally work for a much shorter time than committed employees.

## Footnotes

3 “Formal employees” are permanent employees who generally work for long periods of time, perhaps a long as 30 years.
